# SOX9 Stem-Cell Factor: Clinical and Functional Relevance in Cancer

**DOI:** 10.1155/2019/6754040

**Published:** 2019-04-01

**Authors:** Maribel Aguilar-Medina, Mariana Avendaño-Félix, Erik Lizárraga-Verdugo, Mercedes Bermúdez, José Geovanni Romero-Quintana, Rosalío Ramos-Payan, Erika Ruíz-García, César López-Camarillo

**Affiliations:** ^1^Facultad de Ciencias Químico Biológicas, Universidad Autónoma de Sinaloa, Culiacán, Sinaloa, Mexico; ^2^Laboratorio de Medicina Traslacional y Departamento de Tumores Gastro-Intestinales, Instituto Nacional de Cancerología. CDMX, Mexico; ^3^Posgrado en Ciencias Genómicas, Universidad Autónoma de la Ciudad de México, CDMX, Mexico

## Abstract

Transcriptional and epigenetic embryonic programs can be reactivated in cancer cells. As result, a specific subset of undifferentiated cells with stem-cells properties emerges and drives tumorigenesis. Recent findings have shown that ectoderm- and endoderm-derived tissues continue expressing stem-cells related transcription factors of the SOX-family of proteins such as SOX2 and SOX9 which have been implicated in the presence of cancer stem-like cells (CSCs) in tumors. Currently, there is enough evidence suggesting an oncogenic role for SOX9 in different types of human cancers. This review provides a summary of the current knowledge about the involvement of SOX9 in development and progression of cancer. Understanding the functional roles of SOX9 and clinical relevance is crucial for developing novel treatments targeting CSCs in cancer.

## 1. Introduction

Recently, there has been a growing interest in the study of mechanisms leading to the expression of genes involved in developmental and cell differentiation, since they are related to the presence of a special type of tumor cells with a stemness phenotype dubbed as cancer stem-like cells (CSCs). Stem cells regulatory proteins are now being recognized as potential oncogenes because of their ability to regulate CSCs phenotype and maintenance in tumors of diverse types of cancer. Moreover, it has been well accepted that CSCs are the main driving force behind tumor formation and metastasis [1]. CSCs exhibit diverse cell properties including self-renewal, differentiation capacity, and resistance to apoptosis. Moreover, CSCs are usually resistant to chemotherapy and eventually give rise to recurrence [1, 2]. Sex-determining region Y (SRY)-box 9 protein (SOX9) is a member of the SOX family of transcription factors (TFs) which are developmental regulators that possess high mobility group (HMG) box DNA binding and transactivation domains [3]. It participates in a variety of functions, such as lineage restriction and terminal differentiation, through precise temporal and spatial expression patterns that differ between particular cell types and tissues [4]. SOX9 gene has been implicated in different types of cancer as an oncogene; however, it also may behave as a tumor suppressor [5, 6]. Recent findings have shown that ectoderm- and endoderm-derived tissues continue to express SOX9 in stem cell pools [7] and evidence also suggests that it may regulate CSCs [8–10]. However, detailed mechanisms need to be elucidated. In this review we aim to condense the knowledge about the involvement of SOX9 in the initiation and progression of different types of cancer and to highlight its potential as a clinical biomarker.

## 2. SOX Family of Transcription Factors

SOX family of proteins comprise a group of transcriptional regulators containing a highly conserved HMG domain that was first discovered in SRY protein, a transcription factor involved in mammalian male sex determination [11]. SOX9 is located in chromosome 17 in a 3 Mb region devoid of other protein-coding genes and its expression is complex with individual enhancers directing tissue-specific expression [12]. In general, proteins containing an HMG domain which consists of three *α* helices with 50% or higher amino acid similarity to the HMG are referred to as SOX proteins (SRY-related HMG box). Around 20 related SOX proteins have been identified in humans, and they have been grouped based on the structural homology of regions outside of their conserved HMG boxes [7]. SOX proteins bind to ATTGTT consensus or related sequence motifs through their HMG domain [13]. SOX9 belongs to the SoxE subgroup, and its HMG domain induces significant bending at the consensus-binding motif (A/TA/TCAAA/TG) by forming an L-shaped complex in the minor groove of DNA [7]. Members of the SOXE subgroup share regions of significant homology outside the HMG domain and constitute two additional functional domains: a self-dimerization domain and a transactivation domain at the C-terminus [14]. One current model suggests that dimerization is achieved through cooperative binding between the dimerization domain of one SOXE protein and the HMG box of its partner SOXE protein [15]. SOX protein is subject to posttranslational modifications which alter its nuclear import (phosphorylation and acetylation) and its rate of degradation (ubiquitination and sumoylation) [16]. Individual SOX members within a group share biochemical properties and thus have overlapping functions. Conversely, SOX factors from different groups have acquired distinct biological functions despite recognizing the same DNA consensus motif [4]. Target gene selectivity by different SOX factors can be achieved through differential affinity for particular flanking sequences next to consensus SOX sites, homo- or heterodimerization among Sox proteins, posttranslational modifications of SOX factors, or interaction with other cofactors [4]. The expression of SOX genes is frequently subject to autoregulation or control by other SOX members. SOX is also regulated post-transcriptionally by microRNAs [17]. Furthermore, SOX-dependent regulations intersect with signaling networks such as the sonic hedgehog (Shh) [18], Wnt signaling pathways, in which SOX-Gli and SOX-*β*-catenin interactions are implicated [19]. SOX factors respond to different extracellular signals and interact with a host of intracellular cofactors to control different sets of genes in distinct cell types [20]. Additionally, SOX compete with other transcriptional factors regulating alternative lineages to achieve different cell fates during development. At molecular level, this is often accomplished by directly activating genes that promote their own lineage and repressing genes of alternative lineages [20]. In summary, SOX factors have profound implication in cell fate determination during development, even though recent findings reveal their crucial role in establishing and maintaining stem and progenitor cells [21].

## 3. Role of SOX9 in Human Cancer

SOX9 has been studied from a developmental point of view, particularly during chondrogenesis and male gonad genesis. Nevertheless, recent molecular and functional analyses have elucidated an important role in stem cell biology of mesoderm-, ectoderm-, and endoderm-derived tissues and organs [7]. Importantly, SOX9 maintains both adult stem and progenitor cells with high turnover, as in intestine and hair follicles, and it is crucial for postnatal injury repair in endodermic and ectodermic organs [7]. Remarkably, dysregulation of tissue differentiation pathways and stem cell homeostasis contributed to the initiation and progression of cancer. Experimental and clinical data demonstrated an important role for SOX9 in tumorigenesis since it is overexpressed in a wide range of human cancers where its expression correlated with tumor progression and clinical data (Table 1) [73]. In addition, SOX9 interacted with different transcription factors and exhibited several pro-oncogenic characteristics including promotion of proliferation, senescence inhibition, and neoplastic transformation in collaboration with other oncogenes [73]. COSMIC analysis showed that, from a total of 46,601 unique cancer samples, 572 samples have mutations in SOX9 and the most frequent mutation type is missense substitution (38.81%) of which 113 (33.63%) are C>T transitions. Copy number variations (CNV) gain was reported in 108 unique samples and overexpression was present in 509 samples [74, 75]. The versatility of SOX9 may be explained by a combination of posttranscriptional modifications, binding partners, and the tissue type in which it is expressed [7].

### 3.1. SOX9 Alterations in Hepatocellular Carcinoma

Hepatocellular carcinoma (HCC) is the most common primary liver malignancy. Its genetic complexity relies on interaction between several somatic genomic alterations and diverse etiologies linked to liver diseases by the concerted action of passenger and driver cancer genes. HCC progression is a complex process that implicates accumulative genomic alteration that includes aberrant gene expression, oncogene upregulation, and tumor suppressor downregulation providing favorable conditions for the development of HCC [76–78]. These mechanisms have been associated to several alterations in some important cellular signaling pathways relevant to a therapeutic perspective, such as RAF/MEK/ERK pathway, phosphatidylinositol-3 kinase (PI3K)/AKT/mammalian target of rapamycin (mTOR) pathway, and Wnt/*β*-catenin pathway [79]. Nonetheless, the molecular pathogenesis of HCC is still unclear.

SOX9 regulated by Notch determines the timing and structure of bile duct morphogenesis during liver embryogenesis. Besides, not only during development, but also in the adult organs, SOX9 expression levels appear to be crucial for controlling the cell status of the duct cells [80].* In vitro* analysis has shown that SOX9 expression in HCC cell lines was upregulated in comparison to normal hepatic cell lines; furthermore, it was expressed at higher level in highly metastatic cells lines relative to low metastatic cells. Moreover, downregulation of SOX9 in HCC cell lines decreased invasiveness and migration [81]. Recent studies using SOX9 chromatin immunoprecipitation combined with deep sequencing (ChIP-seq) analysis indicated that SOX9 can activate canonical Wnt/*β*-catenin signaling in HCC endowing stemness features through Frizzled-7 [82]. Besides, the results of a genome-wide transcriptional analysis indicated that TGF*β* and Wnt/*β*-catenin signaling pathways were activated in hepatocholangiocarcinoma (cHCC-CC) [83]. Furthermore, integrative genomics revealed that cHCC-CC shares characteristics of poorly differentiated HCC with stem cell traits and poor prognosis [83]. Interestingly, early biomarkers of biliary commitment such as SOX9, as well as master genes of signaling pathways, which regulate the differentiation of hepatoblasts to cholangiocytes, were induced in cHCC-CC (e.g., TGF*β*, Wnt, and Notch) [83]. SOX9 overexpression was commonly observed in HCC with high tumor stage and tumor grade tissues. Also, the high expression of SOX9 was linked to a significant trend toward both poorer disease-free survival and poorer overall survival [22]. Besides, poor prognosis of HCC patients has been linked with high SOX9 expression independent of the presence of cirrhosis [23].

### 3.2. Role of SOX9 in Breast Cancer

Breast cancer is a complex and heterogeneous disease that includes morphological and molecular different entities. Clinical parameters such as tumor size, lymph node involvement, histological grade, age and the expression of estrogen receptor (ER), progesterone receptor (PGR), and epidermal growth factor receptor 2 (HER2) biomarkers are responsible of its high clinical heterogeneity [84]. Mammary glands contain a small subpopulation of cells with a stem cells activity and it is also known that several TFs play pivotal roles in the establishment of cellular states. SLUG and SOX9 play essential roles in induction and maintenance of tumor initiating capacity in breast cancer cells [58]. In breast tumors, SOX9 expression was higher in comparison to normal mammary tissues, which was associated with an increased proliferation and Ki67 and p53 expression [59–61]. There is also evidence that upregulation of SOX9 affected metastasis and tumorigenesis in breast cancer cells by 5-fold and 40-fold, respectively [9]. Primary tumors with high expression levels of SLUG and SOX9 had a significant lower overall survival rate.

On the other hand, knockout studies have demonstrated that SOX9 was essential for the function of mammary stem/progenitor cell populations. Knockdown of SOX9 resulted in decreased proliferation of mammary stem cells [85]. On the other hand, higher expression of cytoplasmic-SOX9 in breast tumors was significantly associated with ER-status and decreased overall survival [24]. Altogether, these data indicate that cytoplasmic location of SOX9 was directly related with increased proliferation in breast cancer cell lines. Similarly, cytoplasmic SOX9 expression was directly related to neoplastic progression and its nuclear expression was more common in early stages of differentiation [24].

### 3.3. The Importance of SOX9 in Bladder Cancer

Bladder cancer (BC) is the ninth most common malignant disease and the thirteenth most frequent cause of cancer death worldwide. Men are more affected than women (3.2:0.9 ratio) and disease incidence increases with age [86]. In previous studies using biopsies of BC, 75% positive immunostaining of SOX9 was observed in the nucleus of cancer cells and the expression was significantly associated with the advanced pathological grade and clinical stage. However, SOX9 immunostaining in the normal bladder tissues occurred mainly in the cytoplasm and nucleus. These findings could indicate that SOX9 may play a promotive role in the progression of BC [25]. On the other hand, epigenetic changes of SOX9 were associated with the aggressiveness of bladder cancer [87]. Methylation of Sox9 promoter gene was identified in a study of 101 BC samples and it was significantly associated with shorter overall survival. Besides,* in vitro* analyses demonstrated that the expression of SOX9 was aberrantly silenced by CpG island promoter hypermethylation in BC [88]. However, Sun et al. (2009) found a hypermethylated state of SOX9 in only 3/82 (3.7%) cases of BC and 2/15 (13.3%) cases of the control in a Chinese cohort [89]. These results indicated the necessity to further compare the methylation profiles between populations, given the discrepancies in this disease as proposed previously [90].

### 3.4. SOX9 Aberrant Expression in Gastric Cancer

Gastric cancer (GC) is one of the most aggressive malignant tumors worldwide with a high mortality rate, preceded only by lung cancer [91]. Globally, GC is the fourth most common cancer and second leading cause of cancer related mortality with a 5-year overall survival rate less than 25% [86]. SOX9 expression has been found in epithelial cells at the proliferative zone of the normal gastric mucosa and bottom area of the intestinal metaplasia of the stomach. Many tumor cells of type I GC are positive for SOX9 [92, 93]. Ectopic expression of *β*-catenin in AGS and MKN-1 cells induced increased expression of SOX9 [94], whereas its suppression by PPAR*γ* decreased SOX9 expression in MKN-28, SGC-7901, and BGC-823 cells [95]. Gastrokine 1 (GKN1), a tumor suppressor like protein which expression is lost in gastric tumors (including adenoma and cancer) [96], was responsible for decreased SOX9 expression in AGS and MKN-1 cells. Nevertheless, in GC tissues, nuclear expression of SOX9 was closely associated with GKN1 immuno-negativity suggesting that aberrant SOX9 expression by GKN1 inactivation may be involved in the development of sporadic GC as an early event [94].

Additionally, SOX9 overexpression was correlated with lymph node metastasis and advanced tumoral stages of GC, indicating that it is related to tumor progression by promoting invasion and metastasis [97] and with reduced disease-free survival [26].

Moreover, elevated SOX9 levels were associated with resistance to cisplatin in MKN45 and KATO III cells [26] whereas miR-524-5p inhibited SOX9 expression conferring sensitivity to cisplatin-resistant GC cells by targeting its 3`UTR. This is because resistance to cisplatin in GC cells was associated with the expression of miR-524-5p. Thus, overexpression of miR-524-5p in GC cells enhanced their sensitivity to cisplatin and it depends on the downregulating SOX9 [98]. Also, SOX9 has been correlated with the carcinoembryonic antigen-related cell adhesion molecule 1 (CEACAM1). Their coexpression was detected in normal gastric mucosa, hyperplastic polyp, intestinal metaplasia, gastric intraepithelial neoplasia, and adenocarcinoma, showing highly elevated expression from benign proliferative lesions to malignant lesions, suggesting that SOX9 could change CEACAM1 expression patterns, which might promote the tumor progression [99].

*Helicobacter pylori* infection has a very important role in GC. In animal model, histopathological changes such as metaplasia of the gastric mucosa after* H. pylori* infection show increased expression of CD44 and SOX9 dependent on IL-1 signaling, suggesting the participation of SOX9 in gastric carcinogenesis [100]. In humans, the risk of developing GC was higher in individuals infected with cytotoxin-associated gene A (cagA)—positive strains or some vacuolating cytotoxin gene A (vacA) allelic combinations causing the loss of crucial features of epithelial differentiation in gastric cells, leading to transformation and tumor formation [101–103]. In this regard, SOX9 was transcriptionally activated following* H. pylori* infection in GC cell and its silencing resulted in an increase of phospho-histone H3- (p-H3-) proliferative cells and spheres formation ability promoted by bacteria. *β*-catenin-silenced cells also presented a marked reduction in p-H3-positive cells when infected with both strains [26]. Conversely, downregulation of SOX9 by promoter methylation was related to GC progression in Epstein Barr Virus-positive biopsies and infected MKN7 cells. SOX9 methylation was detected in 47% of GCs and correlated with low levels of SOX9 protein. Besides, the rate of methylated SOX9 tumors increased and SOX9 expression gradually decreased through the depth of GC invasion. These data strongly suggested that the decrease of SOX9 expression in advanced GC was related with the epigenetic suppression of SOX9 during tumor invasion [104].

### 3.5. SOX9 Is Involved in Different Types of Pancreatic Cancer

SOX9 regulated by Notch is involved in the maintenance of pancreatic progenitor pools [105]. Furthermore, SOX9 is essential for pancreas development. At the early stages of mouse pancreas development it is expressed in all epithelial cells and its expression is confined to the ductal cells and centroacinar cells as development progresses [80]. Genetic lineage-tracing studies showed that all types of pancreatic epithelial cells including endocrine, acinar, and duct cells express Sox9, suggesting that the all Sox9-expressing cells are a common progenitor of pancreatic epithelial cells [106].

Recently, it was shown that Sox9 is expressed during premalignant and malignant lesions such as mucinous cystic neoplasias (MCNs), intraductal papillary mucinous neoplasias (IPMNs), pancreatic intraepithelial neoplasias (PanINs), and pancreatic ductal adenocarcinoma (PDAC) (Table 2) [34, 107], which is the most common pancreatic cancer and develops from cells lining pancreatic ducts [108]. The evidences suggested that a phenotypic switch converting pancreatic acinar cells to duct-like cells can lead to PanIN [109] and eventually PDAC. Studies about the expression of SOX9 and Hepatocyte Nuclear Factor 6 (HNF6) show that these TFs were expressed in acinar cells. HNF6 induced SOX9 expression, indicating that SOX9 is downstream of HNF6. In acinar-to-ductal metaplasia (ADM), SOX9 was predominantly found in metaplastic cells that displayed duct-like characteristics and it was also found in PanIN [34, 35].

Epidermal growth factor (EGFR) in the PDAC promotes expression of SOX9 as an early event. In this context, pancreatic metaplasia could be also caused by loss of p27 function, a negative regulator of proliferation and a tumor suppressor that inhibits cyclin-CDK activity in the nucleus [110–112]. Besides, K-RAS activation, the earliest known event in pancreatic carcinogenesis [113, 114], may induce p27 mislocalization producing loss of nuclear p27 expression and as a result derepression of SOX9, triggering ADM [36]. The formation of acinar-derived premalignant lesions depends on ectopic induction of SOX9, a ductal gene [115]. Moreover, when it is concomitantly expressed with oncogenic K-RAS, SOX9 accelerated the formation of premalignant PDAC lesions [37]. Furthermore, SOX9 and p-AKT double-positive expression was related with an unfavorable prognosis, high TNM, and distant metastasis in PDAC [38, 116].

Studies using patient-derived tumor organoids with PDAC show that in normal pancreas SOX9 is expressed in the nucleus, while, in the organoids with TP53-mutated (R175H) PDAC, it was expressed in cytoplasm. Clinically, high expression of SOX9 in cytoplasm could be related with a poor disease-free survival (DFS), overall survival, higher tumor grade, and worse disease-specific survival compared to patients with nuclear SOX9 [39]. Emerging evidence suggests that CSCs are exclusively tumorigenic and essential drivers for tumor progression and metastasis [117]. Pancreatic CSCs have been identified and characterized using different surface markers: CD44, CD24, EpCAM, CD133, CXCR4, c-Met, and Aldehyde Dehydrogenase-1a1 (ALDH1) [118–121]. SOX9 has been found expressed in the pancreatic CSCs isolated from PANC1 and HPAC cell lines of pancreatic cancer. This population was more capable of initiating tumors in NOD/SCID xenograft model than the population no-invasive (no-CSCs) [40].

Demethylated SOX9 is found in CSCs and plays a crucial role in the invasion process. Also, NF-*κ*B subunit p65 positively regulates SOX9 expression by directly binding to the SOX9 promoter [40], suggesting that the NF-*κ*B pathway is one of the most activated pathways in pancreatic CSCs. Another important regulator of SOX9 is the glycosyltransferase ST6Gal-I which adds *α*2-6-linked sialic acids to substrate glycoproteins and it is known that its upregulation in cancer cells confers stemness characteristics [41, 122]. Modulating ST6Gal-I expression in pancreatic cancer cells directly altered CSC spheroid growth. In this regard, ST6Gal-I knockdown decreased the levels of SOX9 [41], suggesting that SOX9 expression was regulated by a specific glycosyltransferase, and tumor glycosylation could be a mechanism for functionally shifting cells to a less differentiated, stem-like state.

Interestingly, SOX9 expression in different pancreatic cell lines (PANC-1, Capan-1) was related to stronger chemoresistance to gemcitabine than cells with low SOX9 expression (BxPC-3, MiaPaCa-2). Conversely, SOX9 repression using siRNA recovers the chemosensitivity, affected spheres formation rate, and the proportion of CD44^high^ and CD24^high^ cells. This indicates that the expression of Sox9 plays an important role in chemoresistance by the induction of stemness in pancreatic cancer cells [42].

On the other hand, GLI1, a member of the GLI family of zinc finger transcription factors, is a central regulator of cell fate that is deregulated in diverse tumor types [62–66]. GLI1 signaling impacts multiple cancer-relevant cellular processes, promoting dedifferentiation, the generation of CSCs, tumor progression, and metastasis. GLI1 directly induced the transcription of SOX9 and a positive feedback promoting SOX9-dependent cancer stem cell properties was observed [43].

Epithelial-to-mesenchymal transition (EMT) process is a critical regulator of the CSC phenotype [123, 124]. Tumor growth factor *β* (TGF*β*) induces EMT, promoting cancer cell invasion and metastasis [124]. PANC1 cell line stimulated with TGF*β*1 showed a significant downregulation of SOX9, FOXA2, and GATA4 master genes [44].

Furthermore, p53-/- mice enhanced sphere formation, increased expression of the stemness regulator BMI1 and KLF4 and pancreatic multipotent progenitor markers such as PTF1A, PDX1, CPA1, c-MYC, HNF1B, and SOX9 [45]. These results can be relevant to understand the relationship between p53 and SOX9 and their importance in the acquisition of EMT characteristics in pancreatic cancer.

Microarray analysis demonstrated that another important issue related with cancer and the CSCs was hypoxia, which induced expression of Sox9 in low metastatic cell line FG, whereas in high metastatic cell line L3.6pl it was found constitutively expressed and was not more inducible under hypoxic conditions [46]. Besides, a subset of transcripts related different networks including WNT, CXCR4, retinoic acid, and FAK signaling pathways were also regulated by SOX9 in the aggressive-metastatic cells, but driven by HIF-1*α* in low metastatic cells [46].

Opposite to the oncogenic role of SOX9 in pancreas carcinogenesis studies in tissues corresponding to later stages of tumor development have found downregulation of SOX9 and other master regulators of embryonic development such as GATA4, PDX1, PTF1a, and HNF1b [123, 125–127].

Even though PDAC is the most common type of pancreatic cancer, there are other types of pancreatic tumors with less incidence but more aggressive behavior such as the anaplastic pancreatic cancer (APC), which has been considered a variant of PDAC [67, 68]. There was evidence that PDAC and APC have high expression of Sox9. Also, the expression of proteins related with CSCs and EMT process is higher in APC samples than PDAC, which correlated with aggressiveness of APC [30].

Another type of pancreatic cancer called intraductal papillary mucinous neoplasm (IPMN) is less aggressive than PDAC and APC [47, 128, 129] and exhibits a characteristic expression of SOX9 confined to the lower portions of IPMN which is lost once the neoplasms advance to high-grade dysplasia carcinoma. Cells in the upper portions of IPMN may be, albeit speculative at this point, supplied by the SOX9-positive cells in the lower portions of the neoplasm [47].

Finally, solid pseudopapillary tumor (SPT) is an uncommon type of pancreatic tumor of undetermined origin present especially in children [130]. Studies on samples from pediatric patients with SPT showed that PDX1 and SOX9 were both expressed in the cytoplasm of SPT cells, supporting the hypothesis that tumor cells originate from pancreatic stem cells persisting after the embryonic period [48]. This is relevant since both transcription factors are crucial for pancreatic organogenesis and linked to Wnt/*β*-catenin signaling pathway [105, 131–133].

### 3.6. SOX9 Is Required for Prostate Cancer Initiation

The human prostate is composed of prostatic glands with well-defined basal and luminal epithelial cell layers. Cells within the luminal epithelium have a very low rate of proliferation and express high levels of androgen receptor (AR) [69]. In contrast, basal cells have a higher rate of proliferation, express low or undetectable levels of AR, and are not androgen dependent, playing critical roles in prostate organogenesis, homeostasis, support, and a barrier for the luminal cells. This barrier becomes discontinuous in prostatic intraepithelial neoplasia, which is believed to be a precancerous lesion. The complete loss of the basal cell layer is a defining feature of prostate cancer (PCa) [134].

SOX9 protein was expressed in adult prostate basal epithelium and at the initial stages of bud outgrowth from the urogenital sinus and could play a role in maintaining the committed stem cell phenotype, differentiation, and supporting the overlying luminal epithelium [134–136].

*In vivo* studies showed that SOX9 was highly expressed during fetal prostate development by epithelial cells expanding into the mesenchyme, suggesting that it may contribute to invasive growth in PCa [137]. Besides, SOX9 expression in prostate cancer cells was regulated by Wnt/*β*-catenin signaling, being AR one identified downstream target [134]. In turn, SOX9 positively regulated multiple Wnt pathway genes, including encoded Wnt receptors (frizzled [FZD] and lipoprotein receptor-related protein [LRP] family members) and the downstream *β*-catenin effector TCF4 [70]. Microarray analysis showed that SOX9 was overexpressed in PCa tissues when compared with noncancerous prostate tissues. Also, SOX9 overexpression was found in PCa tissues with higher clinical stage and was related to lower biochemical recurrence-free survival and overall survival rates [138]. When the SOX9 expression was correlated with overexpressed HIVEP3 (human immunodeficiency virus type I enhancer binding protein 3), the patients also exhibited significantly shorter biochemical recurrence-free survival [139].

Some tumor suppressors have been related with SOX9 participation in PCa. Its overexpression in adult mouse prostate epithelia gives rise to an increase in proliferation and induced early high-grade prostate intraepithelial neoplasia lesions when mice are heterozygous for PTEN (phosphatase and tensin homolog deleted on chromosome 10). This study shows that high levels of SOX9 contributed to regulate proliferation within the prostate epithelia and can cooperate with PTEN loss to accelerate prostate neoplasia [140]. Zbtb7a (also known as pokemon) has been recently reported as an oncosuppressor in PCa since it is lost in a subset of human advanced prostate cancer and facilitates the oncogenic activity of SOX9 during prostate tumorigenesis favoring senescence bypass, increase of proliferation rate, apoptosis resistance, and invasive potential [71].

Interestingly, in human castration resistant PCa samples, nMET was remarkably increased. Androgen deprivation induced endogenous nMET which activates SOX9 and promoted cell proliferation and stem-like cell self-renewal in androgen-nonresponsive PCa cells. This indicates that coupregulation of endogenous nMET and SOX9 upon androgen deprivation may activate cell reprogramming to promote transformation and androgen nonresponsive growth [141].

Fusion genes have a very important role in PCa. SOX9 is a critical downstream effector of ERG in TMPRSS2: ERG fusion-positive PCa, and ERG stimulates SOX9 expression by redirecting AR to a cryptic AR-regulated enhancer in the SOX9 gene [142]. Besides, association between TMPRSS2, ERG positive PCa, and rs1859962 at 17q24 has been demonstrated suggesting a molecular mechanism linking the risk region to the ERG pathway where SOX9 is a downstream target. There is also evidence of a positive correlation between SOX9 gene expression and the rs1859962 risk allele in TMPRSS2: ERG positive tumor tissue [143].

Analysis of tissue microarrays of prostate biopsies samples of patient with metastatic castration-resistant PCa shows 18.3% and 87.3% of patients with positive ERG and SOX9 expression, respectively [27]. Besides, ERG and SOX9 are significant risk factors for lower prostate-specific antigen-Progression Free Survival (PFS), Clinical/Radiological-PFS, and Overall Survival after docetaxel treatment suggesting that ERG and SOX9 are potential biomarkers for prediction to docetaxel treatment in mCRPC patients [27]. Conversely, a gradual decrease of SOX9 has been related to a progression to advanced stage, high Gleason grade, and metastatic growth in ERG-positive cancers and these effects were strictly limited to the subset of prostate cancers harboring PTEN deletions [28].

### 3.7. Oncogenic Role of SOX9 in Ovarian Cancer

Ovarian cancer is the most lethal gynecological cancer [144]. Carcinomas of the ovarian surface epithelium correspond to 90% of ovarian malignancies and are classified into four main histological subtypes, which have distinct characteristics regarding genetic abnormalities and specific signaling pathways [144]. Sertoli-Leydig cell tumors, ovarian sex-cord stromal tumors, granulosa cell tumors, and primary ovarian tumors constitute carcinomas in ovary [145].

In normal ovarian development, SOX9 has different expression levels, as wells as roles in comparison to other tissues. During the follicular development in early pre-antral follicles there is not expression of SOX9, but the cells surrounding the developing follicles present nuclear expression. These are very important since they have a participation in the production of collagen or laminin fibers which constructs follicular lamina [146].

Little is known about SOX9 role in ovarian cancer. Nonetheless, higher expression of SOX9 has been found in human Sertoli tumor biopsies coexpressed with BCL-2 and Ki-67, being the last the less expressed in well-differentiated cells [147]. This suggests that apoptotic and proliferative properties depend on tumor differentiation stage. Besides, it is known that hypoxia conditions promote Tubulin Beta 3 (TUBB3) expression through HIF-2*α* and SOX9. High expression of these genes correlates with shorter overall survival in women with ovarian cancer [29].

### 3.8. The Role of SOX9 in Colorectal Cancer

Colorectal cancer (CRC) is a major cause of morbidity and mortality throughout the world [148]. It accounts for over 9% of all cancer incidences [149, 150] and it is the third most common cancer worldwide and the fourth most common cause of death [150]. In most patients, death is not caused by the primary tumor, but rather by its metastasis in other organs and associated complications [151].

Paneth cells are a highly specialized population of intestinal epithelial cells located into the crypts [152]; these cells are critical to the control of the intestinal stem cell (ISC) niche and the intestinal barrier [153, 154]. The function of SOX9 in Paneth cells has not been clarified but* in vitro* studies suggest a role in the control of cell differentiation in the intestinal epithelium [155].* In vitro* and* in vivo* data indicate that Sox9 gene is a transcriptional target of Wnt signaling; this pathway is involved in the regulation of intestinal epithelium homeostasis [156].

Sox9 expression is regulated by TCF4, the main Wnt pathway TF in the intestinal epithelium [155]. This is relevant since mutations in components of the Wnt pathway, including the tumor suppressor APC and *β*-catenin protein, result in stabilization of *β*-catenin, which then continuously interacts with TCF4, leading to constitutive activation of target genes [72]. Moreover, targeted mutations of APC or *β*-catenin are sufficient to initiate tumorigenesis in mouse [157–159], highlighting the importance of the Wnt pathway in the development of cancer.

Recent studies of CRC have found that overexpression of SOX9* in vitro* and* in vivo* was related to several pro-oncogenic properties, including the ability to promote proliferation, inhibit senescence, and collaborate with other oncogenes in neoplastic transformation [6, 49, 50, 73, 160]. The overexpression of SOX9 is related with recurrent distal truncating mutations as frameshift mutations and nonsense mutation in approximately 11% of CRCs; also, SOX9 mutation is strongly associated with coexistent mutant K-RAS and wild type TP53 [49, 50].

Nevertheless, in the particular case of DLD-1 CRC cell line, which has a heterozygous L142P inactivating mutation of SOX9, the restoration of wild type SOX9 expression results in an oncoprotective activity which inhibits cell growth, clonal capacity, and colonosphere formation while decreasing both the activity of the oncogenic Wnt/ß-catenin signaling pathway and the expression of the c-MYC oncogene [6].

**Figure 1:**
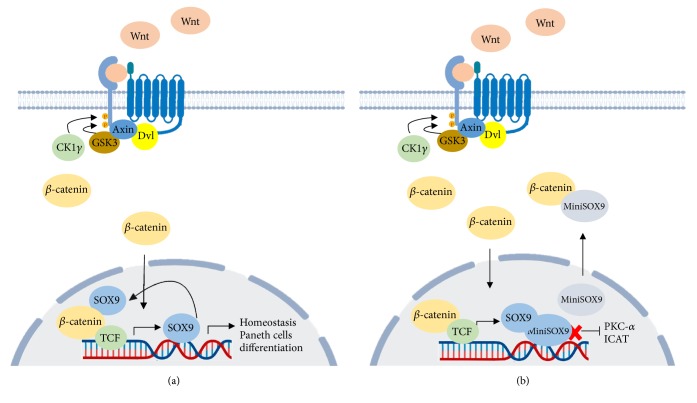
MiniSOX9 has an oncogenic behavior in CRC. (a) In normal conditions canonical Wnt/*β*-catenin pathway triggers SOX9 expression resulting in regulation of differentiation and homeostasis in intestinal epithelium. (b) Truncated version of SOX9, MiniSOX9, accumulates in the nucleus to inhibit SOX9 DNA-binding-dependent transcriptional activity and PKC-alpha expression.

Besides, a truncated version of SOX9 devoid of transactivation domain as a result of retention of the second intron called MiniSOX9 has been discovered in human tumor samples of CRC; this version is expressed at high levels in CRC but it is undetectable in the surrounding healthy tissue. The possible mechanism of MiniSOX9 could be through activation of canonical Wnt target genes and repression of PKC*α* (Figure 1), two features in favor of oncogenic properties [51].

It has been stablished that overexpression of SOX9 in CRC is associated with *β*-catenin activation; however, the largest clinical study on SOX9 expression over 188 primary CRC specimens from a Chinese population shows that it does not present significant correlation between SOX9 and *β*-catenin [31].

SOX9 upregulation is common in colorectal adenoma and cancer and is an independent indicator for an adverse prognosis in CRC [31]. Conversely, low levels of SOX9 at the invasive front of the primary tumor have been shown as an independent predictor of relapse in stage II colon cancer patients (Table 3) [32]. Studies over African Americans CRC cases, diagnosed at earlier ages compared to non-Hispanic withes, have found that SOX9, GATA6, TET1, GLIS1, and FAT1 were differentially hypermethylated in APC mutation-negative CRC; this lack of APC mutation is associated with the early-onset CRC [33].

A recent study about the role and association between SOX9, *β*-catenin, and PPAR*γ* in CRC tissues showed that SOX9 and *β*-catenin were overexpressed whereas PPAR*γ* was downregulated. Treatment with the synthetic PPAR*γ* ligand rosiglitazone induced different changes of SOX9 and *β*-catenin expression and subcellular localization in the colon cancer cell lines Caco2, SW480, HCT116, and HT29. All this data indicated that SOX9, *β*-catenin, and PPAR*γ* expression levels were deregulated in the CRC tissue, and in colon cancer cell lines ligand-dependent PPAR*γ* activation unevenly influences SOX9 and *β*-catenin expression and subcellular localization, suggesting a variable mechanistic role in colon carcinogenesis [52].

In HT29 and HCT116 cell lines of CRC, SOX9 was recruited by NF-Y to the target genes and interacted with NF-Y on CCAAT promoter sequences. Besides, SOX9 is necessary for the function of NF-Y in activating expression of some cell-cycle regulatory gene expressions such as cyclin B1, cyclin B2, cyclin dependent kinase 1, and topoisomerase II *α* [53].

Multiples targets of SOX9 have been described. One of these is FOXK2, a transcription factor which promotes the cell proliferation in samples tissues of CRC [54]. Another important target of SOX9 is S100P; both were coexpressed in CRC and the knockdown of SOX9 expression downregulates S100P expression resulting in reduced invasiveness and metastasis of colon cancer cells by inhibiting the activation of receptor for advanced glycation end products (RAGE)/ERK signaling and EMT [55].

Interestingly, hypoxia induced EMT and SOX9 overexpression in CRC cells. SOX9 was able to migrate to nucleus and upregulated the expression of USP47, a deubiquitinating enzyme [56]. Another way to enhance the EMT by SOX9 is the loss of ZFP36 expression, a tumor suppressor [161]. On the other hand, it was demonstrated that SOX9 levels were higher in metastatic SW620 cell line than in primary CRCs SW480 cell line isolated from the same patient. SOX9 is sufficient and necessary for the acquisition and maintenance of CR-CSCs and metastatic traits, properties linked to transcriptional and post-transcriptional regulation. Finally, SOX9-mediated self-renewal and growth were impaired by the mTOR inhibitor rapamycin [57].

## 4. Clinical Relevance of SOX9 in Cancer

SOX9 has proven its functional role in various aspects of cancer biology. Besides, research on SOX9 has also investigated its importance in the clinic regarding disease prognosis, relapse, and therapy resistance. For instance, SOX9 overexpression was commonly observed in those HCC high tumor stage and tumor grade tissues. Also, the high expression of SOX9 was linked to a significant trend toward both poorer disease-free survival and poorer overall survival [22]. Moreover, poor prognosis of HCC patients has been linked with high SOX9 expression independent of the presence of cirrhosis [23]. In breast cancer, primary tumors that exhibit high expression levels of both SLUG and SOX9 had a significantly lower overall survival rate than the rest of the patients. Thus, SOX9 expression in carcinogenesis and malignity in breast cancer tumors is relevant. Besides, higher expression of cytoplasmic-SOX9 in human breast tumors is significantly associated to ER-status and to decreased overall survival [24]. Previous studies using biopsies of BC have shown that 75% positive immunostaining of SOX9 is observed in the nucleus of cancer cells and this expression is significantly associated with the advanced pathological grade and clinical stage [25]. In the case of GC, SOX9 overexpression has been correlated with lymph node metastasis and advanced tumoral stages, indicating that it is related to tumor progression by promoting invasion and metastasis [97] and with reduced disease-free survival [26]. Analysis of tissue microarrays of prostate biopsies samples of patient with metastatic castration-resistant PCa (mCRPC) showed 18.3% and 87.3% of patients with positive ERG and SOX9 expression, respectively [27]. Besides, ERG and SOX9 are significant risk factors for lower prostate-specific antigen-progression free survival (PFS), Clinical/radiological-PFS, and overall survival after docetaxel treatment suggesting that ERG and SOX9 were potential biomarkers for prediction to docetaxel treatment in mCRPC patients [27]. Conversely, a gradual decrease of SOX9 has been related to a progression to advanced stage, high Gleason grade, and metastatic growth in ERG-positive cancers, and these effects were strictly limited to the subset of prostate cancers harboring PTEN deletions [28].

Clinical relevance of SOX9 in ovarian cancer relies on its coexpression with HIF-2*α* under hypoxia conditions, promoting TUBB3 expression. The combined presence of high TUBB3/SOXn levels is associated with a relevant reduction of PFS and overall survival in women with ovarian cancer [29]. In pancreatic cancer, there is evidence that PDAC and APC have high expression of Sox9. Also, the expression of proteins related with CSCs and EMT process is higher in APC samples than PDAC, which correlates with aggressiveness of APC [30]. SOX9 upregulation is common in colorectal adenoma and cancer and is an independent indicator for an adverse prognosis in CRC [31]. Conversely, low levels of SOX9 at the invasive front of the primary tumor have been shown as an independent predictor of relapse in stage II colon cancer patients [32]. Studies over African Americans CRC cases, diagnosed at earlier ages compared to non-Hispanic withes, have found that SOX9, GATA6, TET1, GLIS1, and FAT1 are differentially hypermethylated in APC mutation-negative CRC; this lack of APC mutation was associated with the early-onset CRC [33].

## 5. Concluding Remarks

Nowadays, we have a solid background about SOX9 function in normal embryonic and adult tissues, and a whole network of regulatory mechanisms depends on and influences SOX9 expression and activity. However, SOX9 expression is also a common characteristic of CSCs. Emerging evidence suggests that CSCs play a crucial role in the development and progression of malignancies. It is already known that SOX9 has an adaptable role since it participates in different steps of cancer progression. For instance, SOX9 is very important in the initiation of pancreatic, gastric, and prostate cancer. Conversely, in bladder and colorectal cancer, SOX9 participates in the progression of the disease, whereas it is correlated to metastasis in breast, gastric, pancreatic, and colorectal cancer. Moreover, SOX9 is clinically relevant as it may contribute in diagnosis, prognosis, and therapeutic among diverse types of cancer. This is because its expression levels and location could be cytoplasmic or nuclear depending on the stage, place, and aggressiveness. Thus, it could serve as a potential biomarker. Besides, SOX9 expression levels are related with chemoresistance in gastric, pancreatic, and colorectal cancer and high expression of SOX9 in several solid tumors is related to poor overall survival, biochemical recurrence-free survival, disease-specific survival, and DFS. Even though SOX9 has a pivotal role in different types of cancer, it has been described as an oncogene and as tumor suppressor. In this regard, it is remarkably important to consider that differences in cell lines, animal models, and populations may cause diverse outcomes. Therefore, more work is needed to study SOX9 participation in Wnt/*β*-catenin and other pathways, including its relationship with other TFs (Table 4) related with stem-cell maintenance in different types of cancer, in order to elucidate additional mechanisms through which it may function. This is specially required for gaining a better understanding of SOX9 roles in normal and disease states to developing novel cancer therapeutic strategies.

## Figures and Tables

**Table 1:** SOX9 expression and functions in human cancers.

Type of cancer	Status of SOX9	Sox9 participation	References
Hepatocellular carcinoma	overexpression	Related whit poor prognosis Related with poor disease free survival Related with poor overall survival	[22, 23]

Breast cancer	overexpression	Promotes proliferation, tumorigenesis and metastasis Related with poor overall survival	[24]

Bladder cancer	overexpression	Promotes tumorigenesis Related with poor overall survival	[25]

Gastric cancer	overexpression	Promotes chemoresistance Related with poor disease free survival	[26]

Prostate cancer	overexpression	Promotes cell proliferation and apoptosis resistance Related with high clinical stage Related with poor relapse free survival Related with poor overall survival	[27]

Prostate cancer	downregulation	Promotes metastasis Related with advanced clinical stage Related with EGR-positive tumors	[28]

Ovarian cancer	overexpression	Its coexpression with HIF-2*α* induces the expression of TUBB3 which is related with poor overall survival	[29]

Pancreatic cancer	overexpression	Promotes chemoresistance	[30]

colorectal cancer	overexpression	Promotes cell proliferation, senescence inhibition and chemoresistance	[31–33]

**Table 2:** Sox9 is expressed during premalignant and malignant lesions in pancreatic cancer.

Lesions	Model	Status of SOX9	Effects	References
Acinar-to-ductal metaplasia	Mice	Overexpressed	HNF6 induces Sox9 expression, which is characteristic of ADM in humans	[34, 35]

Acinar-to-ductal metaplasia	Mice	Overexpressed	Aberrant expression of p27 induces the nuclear expression of Sox9	[36]

Mucinous cystic neoplasias, intraductal papillary mucinous neoplasias, pancreatic intraepithelial neoplasias and pancreatic ductal adenocarcinoma	Mice	Overexpressed	SOX9 and Kras co-expression is associated with PDAC initiation	[37]

Pancreatic ductal adenocarcinoma	88 tumors samples of PDAC	Overexpressed	Sox9 and p-Akt double-positive expression is related with an unfavorable prognosis, high TNM and distant metastasis	[38]

Pancreatic ductal adenocarcinoma	Patient-derived tumor organoids with PDAC	Overexpressed in cytoplasm	High expression of Sox9 in cytoplasm is related with a poor DFS, OS, higher tumor grade and worse disease-specific survival compared to patients with nuclear Sox9 expression	[39]

Pancreatic cancer	PANC1 and HPAC cell lines of pancreatic cancer	Overexpressed	Sox9 is highly expressed in pancreatic CSCs. Moreover, NF-*κ*B subunit p65 positively regulates SOX9	[40]

Pancreatic ductal adenocarcinoma	HD3 colon cancer cells	Overexpressed	ST6Gal-I induces expression of Sox9, promoting stem-like cell properties	[41]

Pancreatic cancer	PANC-1, Capan-1, BxPC-3, MiaPaCa-2 cell lines of pancreatic cancer	Expression depending on chemoresistance	High level of Sox9 is related to stronger chemoresistance to Gemcitabine	[42]

Pancreatic ductal adenocarcinoma	HPDE cell line of PDAC	Overexpressed	GLI1 induces the transcription of Sox9 promoting stem cell properties	[43]

Pancreatic cancer	PANC1 cell line of pancreatic cancer	Downregulated	Induction of EMT with TGF-*β* results in low levels of SOX9, FOXA2, and GATA4	[44]

Pancreatic cancer	Mice	Overexpressed	p53-/- mice enhanced sphere formation, increased expression of the stemness regulator Bmi1 and Klf4, and pancreatic multipotent progenitor markers as Ptf1a, Pdx1, Cpa1, c-myc, Hnf1b and Sox9	[45]

Pancreatic cancer	FG and L3.6pl cell lines of pancreatic cancer under hypoxia	Overexpressed	Under hypoxia conditions, FG cell line expresses high levels of Sox9 and L3.6pl. Besides, WNT, CXCR4, retinoic acid, and FAK signaling pathways are regulated by Sox9 in L3.6pI	[46]

Pancreatic ductal adenocarcinoma and anaplastic pancreatic cancer	6 APC patients and 53 PDAC patients	Overexpressed	PDAC and APC have high expression of Sox9, being the expression of proteins related with CSCs and EMT process higher in APC samples than PDAC	[30]

Intraductal papillary mucinous neoplasm	19 IPMN cases	Overexpressed	SOX9-positive cells were confined to the lower portions of the papillary structures of IPMN	[47]

Solid pseudopapillary tumor	8 samples of SPT	Overexpressed	PDX1 and Sox9 are both expressed in the cytoplasm of SPT cell	[48]

**Table 3:** SOX9 roles in CRC as oncogene and tumor suppressor.

Model	Status of SOX9	Effects	References
353 tumors samples of CRC	SOX9 mutated and WT are overexpressed	Truncating SOX9 mutations are associated with SOX9 overexpression, KRAS mutation, and TP53 wild type	[49, 50]

DLD-1 cell line of CRC	Loss of SOX9 transcriptional activity by L142P mutation	Restoration of wild type SOX9 expression inhibits cell growth, clonal capacity and colonosphere formation; besides, the expression of the c-MYC and the activity of Wnt/ß-catenin signaling pathway are affected	[6]

17 tumors samples of CRC	High levels of SOX9 and MiniSOX9	Overexpression of MiniSOx9 is found in CCR tissues whereas SOX9 is also expressed in normal and adjacent tissues	[51]

188 tumors samples of CRC from Chinese population	Overexpressed	Does not show significant correlation between SOX9 and *β*-catenin	[31]

144 primary tumors from patients diagnosed in stage II CRC	Downregulated	Low levels of SOX9 have been shown as an independent predictor of relapse in stage II colon cancer patients	[32]

45 tumors samples of CRC from African Americans population	Hypermethylated	SOX9, GATA6, TET1, GLIS1, and FAT1 are differentially hypermethylated in APC-negative CRC	[33]

CaCo2, SW480, HCT116 and HT29 cell lines of CRC	Overexpressed	The synthetic PPAR*γ* ligand rosiglitazone induces changes of SOX9 and *β*-catenin expression and subcellular localization	[52]

HT29 and HCT116 cell lines of CRC	Cofactor of NF-Y	SOX9 is necessary for the function of NF-Y in activating expression of cyclin B1, cyclin B2, cyclin dependent kinase 1 and topoisomerase II *α*	[53]

HCT116, SW480, SW620, DLD-1 cell lines of CRC	Overexpressed	Sox9 promotes proliferation through FOXK2	[54]

HCT116 cell line of CRC	Overexpressed	Sox9 promotes invasiveness and metastasis in CRC through S100P	[55]

CCD 841 CoN, DLD-1, HCT-116, and HT-29 cell lines of CRC under hypoxia	Overexpressed	Sox9 upregulates the expression of USP47 promoting EMT under hypoxia	[56]

SW620 and SW480 cell lines of CRC	Overexpressed	SOX9 mediates the acquisition and maintenance of CR-CSCs	[57]

**Table 4:** Role of SOX9 and associated transcription factors in diverse types of cancer.

Type of cancer	TF	Effects	References
Breast cancer	SLUG (SNAI2)	Induction and maintenance of tumor initiating capacity in breast cancer cells	[58]

Breast cancer	TP53	Increased proliferation	[59–61]

Pancreatic cancer	HNF6 (ONECUT1)	Produces ectopic expression of Sox9 in acinar cells converting them in ductal cells	[34, 35]

Pancreatic cancer	NF-*κ*B	NF-*κ*B subunit p65 positively regulates SOX9 expression by directly binding to the SOX9 promoter	[40]

Pancreatic cancer	GLI1	Induces the transcription of SOX9	[62–66]

Pancreatic cancer	PDX1	Co-expressed in the cytoplasm with Sox9 in solid pseudopapillary tumors	[67, 68]

Prostate cancer	AR	Downstream target of SOX9	[69]

Prostate cancer	TCF4	It is positively regulated by SOX9	[70]

Prostate cancer	ZBTB7A (POKEMON)	It is lost in advance prostate cancer facilitating the oncogenic activity of SOX9	[71]

Ovarian cancer	HIF2A (EPAS1)	Hif-2*α* and Sox9 promote TUBB3 expression. High expression of TUBB3 and SOX9 correlates with shorter overall survival	[29]

Colorectal cancer	TCF4	Positively regulates SOX9	[72]

Colorectal cancer	PPAR*γ*	In cell lines ligand-dependent PPAR*γ* activation unevenly influences SOX9 and *β*-catenin expression and subcellular localization, suggesting a variable mechanistic role in colon carcinogenesis	[52]

Colorectal cancer	NF-Y	SOX9 is necessary for the function of NF-Y in activating expression of cyclin B1, cyclin B2, cyclin dependent kinase 1 and topoisomerase II *α*	[53]

Colorectal cancer	FOXK2	Is a SOX9 target and promotes proliferation	[54]
